# RPT-Fusion: A Time-Lag-Aware Quality-Adaptive Radar–Camera Fusion Framework for Water-Surface Object Detection

**DOI:** 10.3390/s26185860

**Published:** 2026-09-16

**Authors:** Yabin Xu, Sujie Zhan, Junnan Yang, Weiming Wang, Honghua Chen

**Affiliations:** 1 School of Mechanical Engineering, Zhejiang Sci-Tech University, Hangzhou 310018, China; yabinxu@zstu.edu.cn (Y.X.);; 2 School of Science and Technology, Hong Kong Metropolitan University, Hong Kong, China; wmwang@hkmu.edu.hk; 3 School of Data Science, Lingnan University, Hong Kong, China

**Keywords:** water-surface object detection, radar–camera fusion, 4D millimeter-wave radar, probabilistic radar prior, temporal reliability modeling, quality-adaptive fusion

## Abstract

Water-surface object detection remains challenging under strong reflections, wave disturbances, adverse weather, low illumination, and distant or small targets, which can severely degrade or obscure discriminative visual cues. Although radar–camera fusion provides complementary geometric and motion information, sparse and uncertain radar observations may introduce unreliable cues, while camera–radar temporal asynchrony can further reduce cross-modal spatial consistency. To address these issues, this paper proposes Radar Prior and Time-Lag-Aware Quality-Adaptive Fusion (RPT-Fusion), a radar–camera fusion framework that preserves the camera as the primary semantic modality while treating radar as a reliability-controlled auxiliary source. Sparse 4D radar measurements are transformed into probabilistic occupancy, density, velocity, and reliability priors, with direction-dependent spatial uncertainty represented through anisotropic radar-prior modeling. Local, global, and temporal quality estimates are then jointly used to regulate radar contributions during multi-scale feature fusion. In particular, the measured camera–radar time lag is explicitly incorporated into both radar-prior construction and feature-level reliability control. Experiments on WaterScenes show that RPT-Fusion achieves 92.31% mAP50 at an Intersection-over-Union (IoU) threshold of 0.50 and 68.23% mAP50–95 averaged over IoU thresholds from 0.50 to 0.95, outperforming WS-DETR by 0.82 and 3.69 percentage points, respectively. Ablation experiments verify the contributions of radar-prior modeling, quality-adaptive fusion, and temporal quality, while repeated-training experiments show low performance variability. Time-lag analysis reveals condition-dependent benefits, with the largest observed improvement of 1.15 percentage points occurring when the camera–radar offset reaches or exceeds 20 ms. Radar-frame-dropping experiments further show that mAP50 decreases from 92.31% to 91.54% when radar observations are progressively removed, indicating that the camera-centric pathway retains a substantial detection capability under incomplete radar observations. These results demonstrate the effectiveness of reliability-controlled radar assistance for robust radar–camera object detection in complex water-surface environments.

## 1. Introduction

Water-surface object detection under complex maritime conditions is a fundamental perception task for unmanned surface vehicles (USVs), intelligent vessels, and autonomous navigation systems. Reliable environmental perception directly supports autonomous navigation, maritime surveillance, collision avoidance, and other safety-critical operations [1,2,3,4]. However, water-surface environments are highly dynamic and visually complex. Specular reflection, wave disturbance, rain, fog, low illumination, and complex backgrounds can weaken object appearance and boundary cues, while safety-critical targets often appear at long distances with small image sizes [5,6,7,8,9]. These factors make robust camera-based detection difficult, particularly for distant and small targets whose visual features are weak and easily disturbed by complex water-surface conditions.

Recent vision-based water-surface detectors mainly improve feature extraction, multi-scale representation, attention mechanisms, lightweight deployment, and small-object detection. Zhao et al. [5] introduced attention mechanisms and dynamic task-aligned sample assignment for water-surface detection, while Wang and Zhao [6] improved YOLOv8 for targets with large-scale variations and complex water-surface interference. Lightweight and multi-scale designs have also been investigated for USV and maritime perception [3,4,10,11,12,13]. More recent methods have further improved maritime detection under glare, low illumination, complex backgrounds, and small-target conditions through water-aware enhancement and strengthened feature pyramids [7,8,9]. Although these methods improve visual feature representation, their performance still fundamentally depends on image appearance and therefore remains vulnerable when complex water-surface conditions degrade visual cues.

Radar–camera fusion provides an alternative way to improve perception robustness by exploiting the complementary sensing characteristics of the two modalities. Cameras provide rich appearance and semantic information, whereas millimeter-wave radar offers range, Doppler velocity, and geometric measurements, and is less sensitive to illumination and some adverse weather conditions [14,15]. Such complementary sensing characteristics are particularly valuable in complex water-surface scenes, where visual information may be degraded by reflection, fog, weak illumination, or distant target appearance. WaterScenes demonstrates that 4D radar–camera fusion can improve water-surface perception, particularly under challenging illumination and weather conditions [1]. Recent radar–camera fusion methods have explored projected radar representations, feature-level interaction, adaptive attention, and multi-level fusion [16,17,18,19]. However, radar observations are themselves sparse and uncertain, and their quality may vary due to clutter, weak echoes, target reflectivity, sensing geometry, and measurement distance. Consequently, directly introducing raw radar points or unconstrained radar features may propagate unreliable information into the detector [14,15,18].

Another important issue in complex water-surface perception is camera–radar temporal asynchrony. Practical cameras and radars may operate at different sampling frequencies or timestamps, and target or platform motion during the acquisition interval can make projected radar observations inconsistent with the corresponding camera frame. Such temporal inconsistency may further reduce the effectiveness of radar assistance, especially when the visual target region is small or weak. Recent studies on asynchronous multimodal sensing have shown that delayed sensing information can increase spatial uncertainty and degrade cross-modal consistency [20,21,22]. Nevertheless, existing water-surface radar–camera methods mainly focus on radar representation and spatial fusion, while the measured camera–radar time difference is rarely incorporated as an explicit reliability factor during both radar representation and feature fusion [1,14].

Accordingly, the objective of this paper is to improve robust object detection in complex water-surface environments by developing a radar–camera fusion framework that explicitly models radar reliability and camera–radar temporal inconsistency, while preserving visual semantic dominance and selectively exploiting complementary radar information. Particular attention is given to distant and small targets, whose visual cues are more easily degraded under challenging water-surface conditions.

To achieve this objective, we propose Radar Prior and Time-Lag-Aware Quality-Adaptive Fusion (RPT-Fusion), a radar–camera fusion framework for water-surface object detection. The core idea is to use radar as a quality-controlled auxiliary source rather than as an equally dominant modality. Sparse 4D radar observations are first converted into occupancy, density, velocity, and reliability priors to provide structured spatial, motion, and confidence information. A camera-centric primary-auxiliary architecture then preserves RGB features as the main semantic pathway, while radar features are selectively introduced through quality-adaptive fusion. Local and global quality terms characterize radar reliability at spatial and scene levels, while the temporal quality term explicitly transforms camera–radar time lag into a reliability cue. This temporal factor influences both radar prior construction and feature-level fusion, allowing unreliable or temporally inconsistent radar observations to be adaptively suppressed under complex water-surface conditions. Compared with direct concatenation or symmetric multimodal fusion, RPT-Fusion explicitly separates radar representation, reliability estimation, and contribution control. The measured temporal offset is further coupled with both probabilistic radar prior construction and quality-adaptive feature fusion, forming a unified reliability-control process that enables radar to provide complementary information without interfering with the primary visual representation.

The main contributions are summarized as follows:

- A time-lag-aware dual-level quality modeling strategy is proposed to explicitly incorporate the measured camera–radar temporal offset into radar reliability modeling. The resulting temporal quality jointly regulates radar prior construction and feature-level fusion, reducing the influence of temporally inconsistent radar observations in complex water-surface scenes.
- A probabilistic radar prior representation is proposed to encode sparse 4D radar observations into occupancy, density, velocity, and reliability maps, providing structured spatial, motion, and reliability cues for robust water-surface object detection.
- A camera-centric quality-adaptive fusion mechanism is designed to preserve visual semantic dominance while adaptively regulating auxiliary radar contributions through local, global, and temporal quality estimation, improving the reliability of multimodal perception under complex water-surface conditions.

## 2. Related Work

### 2.1. Water-Surface Datasets and Vision-Based Detection

Public datasets have substantially promoted the development of water-surface perception. MaSTr1325, MODS, USVInland, FloW, and LaRS cover representative tasks including USV obstacle detection, inland-waterway perception, floating-object detection, and maritime scene understanding [23,24,25,26,27]. These datasets contain diverse water-surface conditions involving reflections, waves, changing illumination, complex backgrounds, and targets at different scales. WaterScenes further provides synchronized RGB images, 4D millimeter-wave radar measurements, timestamps, and annotations for multiple water-surface perception tasks, making it particularly suitable for studying multimodal perception under complex maritime conditions [1].

Vision-based water-surface detection methods mainly improve general object detectors through lightweight architectures, attention mechanisms, multi-scale feature representation, improved sample assignment, and task-specific modules [3,5,6,10,28]. Recent studies have further focused on detection under glare, low illumination, complex backgrounds, and small-target conditions. These methods improve visual representation and detection efficiency, but their effectiveness remains strongly dependent on image appearance. In complex water-surface environments, visual cues may be weakened by reflection, fog, illumination changes, or distant target appearance, making complementary sensing information valuable for improving detection robustness.

### 2.2. Radar Representation for Water-Surface Perception

Millimeter-wave radar provides complementary range, Doppler velocity, and spatial information, and is less dependent on visual appearance than cameras. It has therefore been increasingly introduced into water-surface perception systems [29,30,31,32]. Cheng et al. investigated camera–radar fusion for small-object detection on water surfaces [29], while Zhu et al. further explored radar-assisted visual detection for small water-surface targets [30]. WaterScenes also demonstrates the potential of 4D radar to complement visual perception under challenging illumination and weather conditions [1].

Existing radar–camera methods adopt different radar representations, including projected radar points, image-plane heatmaps, and learned radar features [14,33,34,35]. These representations enable radar information to participate in visual detection, but sparse radar measurements are not uniformly reliable. Radar observations may be affected by clutter, weak echoes, multipath effects, target reflectivity, sensing geometry, and measurement distance. Such uncertainty becomes particularly important in complex water-surface scenes, where useful target responses may coexist with strong reflections and environmental interference. Representing heterogeneous radar measurements with a single undifferentiated map or unconstrained feature stream may therefore obscure the physical meaning and reliability of different radar cues.

This motivates a structured radar representation that separately describes spatial occupancy, local point support, motion information, and observation reliability before radar information is introduced into the visual pathway. Such a representation provides a more interpretable, reliable, and practical basis for exploiting radar under complex water-surface conditions.

### 2.3. Quality-Adaptive Radar–Camera Fusion

Radar–camera fusion can be performed at the input, feature, or decision level. Input-level fusion is straightforward but provides limited control over unreliable radar responses, whereas feature-level fusion enables stronger cross-modal interaction but may also propagate noisy radar information into the visual representation. Recent methods such as Achelous and ASY-VRNet employ vision–radar fusion for multi-task water-surface perception [31,32], while WS-DETR introduces radar–camera fusion into a transformer-based detector for water-surface object detection [36]. More general radar–camera methods, including CenterFusion, CRAFT, and RCFusion, further demonstrate the effectiveness of intermediate cross-modal feature interaction [33,34,35]. Although these approaches demonstrate the benefits of radar–camera fusion, their main emphasis is generally placed on how to establish cross-modal interaction.

In complex water-surface scenes, however, the usefulness of radar information may vary substantially across spatial locations and sensing conditions. Reliable target echoes and strong clutter can coexist within the same frame, making a single scene-level reliability estimate insufficient for characterizing spatially heterogeneous radar quality. Local reliability modeling is therefore required to distinguish useful radar responses from unreliable regions, while complementary global context provides scene-level information about the overall radar feature state. Combining both levels of information enables radar contribution to be regulated according to its actual reliability rather than being injected uniformly into the visual representation. Since camera images remain the primary source of category-level appearance and semantic information, a camera-centric fusion architecture is particularly suitable for water-surface detection. Such a design preserves the visual pathway as the main semantic representation while treating radar as an auxiliary source whose contribution is adaptively controlled according to estimated quality.

### 2.4. Time-Lag-Aware Multimodal Fusion

Most radar–camera fusion studies assume that measurements from different sensors are sufficiently synchronized. In practical perception systems, however, cameras and radars often operate at different acquisition frequencies and timestamps. Existing water-surface fusion studies mainly focus on radar projection, radar representation, and spatial feature interaction [14,29,30,31,32,33,34,35], whereas temporal inconsistency between sensor observations has received relatively less attention. Even when radar–camera spatial calibration is accurate, target or platform motion during the acquisition interval can change the scene between the radar and camera timestamps. Consequently, a radar observation that is geometrically valid at the radar acquisition time may become less consistent with the corresponding region in the camera frame. This effect can be particularly relevant when visual targets are distant or occupy small image regions.

Recent studies on asynchronous multimodal sensing have begun to investigate temporal synchronization, delayed observations, and the uncertainty introduced by temporally stale sensing information [20,21,22]. Fan et al. [20] model the effect of delayed visual sensing on spatial uncertainty in highly mobile multimodal systems, highlighting that temporal freshness can directly influence the reliability of spatial sensing information. Related radar–camera studies further demonstrate that temporal inconsistency constitutes an additional source of uncertainty beyond spatial calibration error [21,22]. However, existing water-surface radar–camera fusion methods rarely use the measured camera–radar time difference as an explicit reliability variable. In particular, temporal information is seldom coupled with both radar representation and feature-level radar contribution control within a unified framework. Therefore, temporal offset should be treated not merely as a synchronization artifact or an additional input feature but as a reliability factor that determines how asynchronous radar observations should be represented and how strongly they should influence multimodal perception. This gap motivates the time-lag-aware reliability modeling adopted in this work.

## 3. Proposed Method

### 3.1. Overall Architecture

Complex water-surface environments pose substantial challenges to robust radar–camera object detection. Visual observations may be degraded by strong reflections, wave disturbances, adverse weather, low illumination, and complex backgrounds, while distant and small targets often provide only weak appearance cues. Although 4D millimeter-wave radar can provide complementary geometric and motion information, radar observations are sparse and may contain clutter, weak echoes, and spatially varying uncertainty. In addition, asynchronous acquisition between the camera and radar can further reduce the consistency between projected radar observations and their corresponding visual regions.

**Figure 1:**
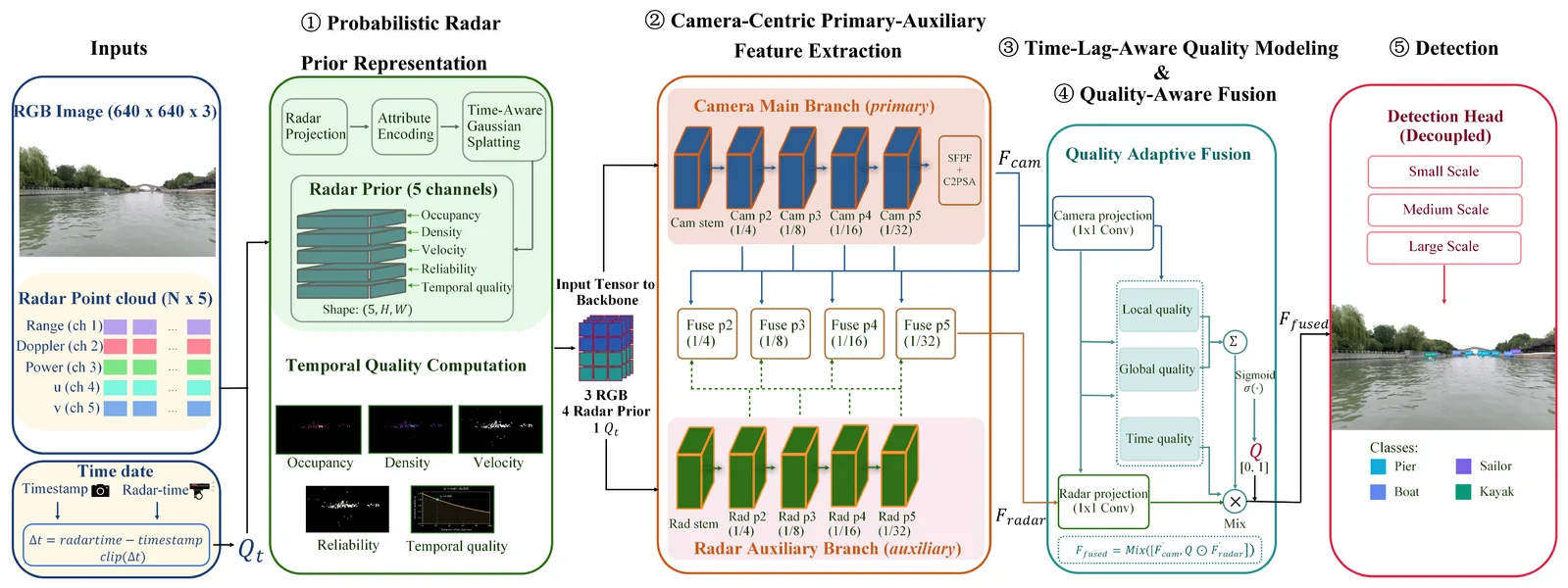
Overall architecture of the proposed RPT-Fusion framework.

To address these challenges, we propose RPT-Fusion, a Radar Prior And Time-Lag-Aware Quality-Adaptive Fusion framework for robust object detection in complex water-surface environments. As shown in Figure 1, RPT-Fusion follows a camera-centric primary-auxiliary architecture and consists of four main stages: probabilistic radar prior representation, camera-centric primary-auxiliary feature extraction, time-lag-aware quality modeling, and local–global–temporal quality-adaptive fusion.

Given a camera image $I_{t_{c}}$ and a radar observation $R_{t_{r}}$ acquired at timestamps $t_{c}$ and $t_{r}$, respectively, the detection task is formulated as

(1) $$\hat{Y}=D_{\theta }\left(I_{t_{c}},R_{t_{r}},\Delta t\right),\;\Delta t=t_{r}-t_{c}$$

where $\hat{Y}$ denotes the predicted object categories and bounding boxes; $D_{\theta }\left(\cdot \right)$ denotes the proposed parameterized radar–camera detection framework that maps the camera observation, the radar observation, and their temporal offset to the detection output; and θ represents its learnable parameters.

The overall data flow is designed to address the major limitations identified in Section 2. Sparse radar observations are first transformed into structured probabilistic priors instead of being directly injected into the detector. The RGB image is processed by the primary camera branch, whereas the radar representation is encoded by a lightweight auxiliary branch to obtain paired multi-scale features. Meanwhile, the measured camera–radar temporal offset is explicitly modeled as both spatial uncertainty and observation reliability. At the prior level, temporal inconsistency adjusts the spatial support and confidence of projected radar observations. At the feature level, temporal reliability further participates in adaptive radar contribution control. Finally, local, global, and temporal quality terms jointly generate a scale-specific gate that selectively regulates radar features before fusion with the primary visual representation.

### 3.2. Probabilistic Radar Prior Representation

As discussed in Section 2.2, sparse radar measurements cannot be assumed to be uniformly reliable in complex water-surface environments. Their spatial distribution, local support, motion information, and observation confidence may vary substantially due to clutter, weak echoes, sensing distance, target reflectivity, and environmental interference. Directly introducing such heterogeneous radar responses into the detector may therefore limit both robustness and interpretability. To obtain a structured auxiliary representation, RPT-Fusion converts each radar frame into four probabilistic priors: occupancy, density, velocity, and reliability. For the *i*-th valid radar observation, the range $r_{i}$, Doppler velocity $d_{i}$, echo power $p_{i}$, and image-plane coordinates $\left(u_{i},v_{i}\right)$ are used. The projected coordinates satisfy

(2) $$\lambda_{i}\left[\begin{array}{c} u_{i}\\ v_{i}\\ 1 \end{array}\right]=K\left(R_{rc}P_{i}^{r}+t_{rc}\right)$$

where $P_{i}^{r}$ denotes the radar point in the radar coordinate system, $R_{rc}$ and $t_{rc}$ denote the radar-to-camera extrinsic parameters, K denotes the camera intrinsic matrix, $\lambda_{i}$ is the projective scale factor, and $\left(u_{i},v_{i}\right)$ denotes the resulting two-dimensional radar-point location on the camera image plane after radar-to-camera coordinate transformation and perspective projection. In our implementation, the projected image coordinates $\left(u_{i},v_{i}\right)$ provided by WaterScenes are directly read from the radar data files rather than recomputed online.

This projection establishes the geometric correspondence between the three-dimensional radar measurement space and the two-dimensional camera image plane. Specifically, the radar-to-camera rigid transformation first expresses each radar observation in the camera coordinate system, after which the camera intrinsic model maps the transformed point onto the image plane through perspective projection. The resulting correspondence provides the spatial basis for constructing image-aligned radar priors and for subsequently integrating radar information with visual features. It should be noted that this projection accounts for spatial calibration only; possible spatial inconsistency caused by asynchronous camera–radar acquisition is modeled separately through the time-lag-aware reliability mechanism described in Section 3.4. The radar attributes are normalized as

(3) $$\begin{array}{cc} {\tilde{r}}_{i} & =\mathrm{clip}\left(\frac{r_{i}}{r_{\max}},0,1\right)\\ {\tilde{d}}_{i} & =\mathrm{clip}\left(\frac{d_{i}}{d_{\max}},-1,1\right)\\ {\tilde{p}}_{i} & =\mathrm{clip}\left(\frac{p_{i}}{p_{\max}},0,1\right) \end{array}$$

where $r_{\max}$, $d_{\max}$, and $p_{\max}$ denote the normalization scales for range, Doppler velocity, and echo power, respectively, and clip(x,a,b)=min{max(x,a),b} restricts *x* to the interval [a,b]. To better characterize the spatial uncertainty of sparse radar observations under complex water-surface sensing conditions, the conventional isotropic Gaussian representation is replaced with a range-dependent anisotropic formulation. This design is motivated by the inherently direction-dependent resolution characteristics of 4D radar, for which the range and angular dimensions exhibit different sensing resolutions. Rather than enforcing identical diffusion in the two image-plane directions, separate horizontal and vertical uncertainty scales are introduced as a lightweight image-plane approximation of this anisotropic sensing behavior. The horizontal and vertical base uncertainty scales of the *i*-th radar observation are defined as

(4) $$\sigma_{u,i}^{0}=\sigma_{u,b}+\alpha_{u}{\tilde{r}}_{i},\;\sigma_{v,i}^{0}=\sigma_{v,b}+\alpha_{v}{\tilde{r}}_{i}$$

where $\sigma_{u,b}$ and $\sigma_{v,b}$ denote the horizontal and vertical base scales, while $\alpha_{u}$ and $\alpha_{v}$ control their range-dependent expansion rates. The range-dependent terms allow the image-plane spatial support to increase with sensing distance, reflecting the growing projection uncertainty associated with angular sensing uncertainty. Let

(5) $$\mu_{i}=\left[\begin{array}{c} u_{i}\\ v_{i} \end{array}\right],\;\Sigma_{i}^{0}=\left[\begin{array}{cc} {\left(\sigma_{u,i}^{0}\right)}^{2} & 0\\ 0 & {\left(\sigma_{v,i}^{0}\right)}^{2} \end{array}\right]$$

where $\mu_{i}$ denotes the projected radar-point center and $\Sigma_{i}^{0}$ represents its base image-plane uncertainty. The diagonal covariance therefore preserves direction-dependent spatial uncertainty without imposing identical horizontal and vertical diffusion. It should be noted that this formulation is an image-plane approximation motivated by the anisotropic resolution characteristics of the radar hardware, rather than an exact physical covariance obtained by directly projecting the radar beamwidth and range-bin dimensions through the complete radar–camera geometry. The intrinsic reliability of each radar observation is estimated from echo strength and sensing distance, as follows:

(6) $${\hat{w}}_{i}^{\mathrm{rel}}={\tilde{p}}_{i}\exp\left(-\beta_{r}{\tilde{r}}_{i}\right)$$

where $\beta_{r}$ controls range-dependent reliability attenuation. Temporal inconsistency further modifies both $\Sigma_{i}^{0}$ and ${\hat{w}}_{i}^{\mathrm{rel}}$. Using the temporally adjusted kernel $K_{i}^{t}$ and reliability weight $w_{i}^{t}$ derived in Section 3.4, the final radar priors are constructed as

(7) $$\begin{array}{cc} M_{\mathrm{occ}}\left(z\right)\; & =1-\exp\left(-\sum_{i=1}^{N}K_{i}^{t}\left(z\right)\right)\\ M_{\mathrm{den}}\left(z\right)\; & =\mathrm{clip}\left(\frac{\log\left(1+\sum_{i=1}^{N}K_{i}^{t}\left(z\right)\right)}{\log(1+\eta )},0,1\right)\\ M_{\mathrm{vel}}\left(z\right)\; & =\frac{1}{2}\left[1+\frac{\sum_{i=1}^{N}{\tilde{d}}_{i}{\tilde{p}}_{i}K_{i}^{t}\left(z\right)}{\sum_{i=1}^{N}{\tilde{p}}_{i}K_{i}^{t}\left(z\right)+\epsilon }\right]\\ M_{\mathrm{rel}}\left(z\right)\; & =1-\exp\left(-\sum_{i=1}^{N}w_{i}^{t}K_{i}^{t}\left(z\right)\right) \end{array}$$

where $z={\left[x,y\right]}^{T}$, *N* denotes the number of valid radar observations, η controls density normalization, and ϵ is a small constant for numerical stability. The logarithmic compression in $M_{\mathrm{den}}$ is used to reduce the dynamic range of accumulated local radar support, preventing regions with densely overlapping radar responses from dominating the representation while preserving relative differences in low- and medium-density regions. The occupancy prior describes radar-supported image regions, the density prior characterizes local radar-point support, the velocity prior encodes Doppler motion information, and the reliability prior represents confidence after both intrinsic and temporal reliability regulation. For clarity, $K_{i}^{t}\left(z\right)$ in Equation (7) refers to the same temporally adjusted anisotropic radar kernel explicitly defined in Equation (13).

The complete radar representation is written as

(8) $$M_{\mathrm{radar}}=\left[M_{\mathrm{occ}},M_{\mathrm{den}},M_{\mathrm{vel}},M_{\mathrm{rel}}\right]$$

**Figure 2:**
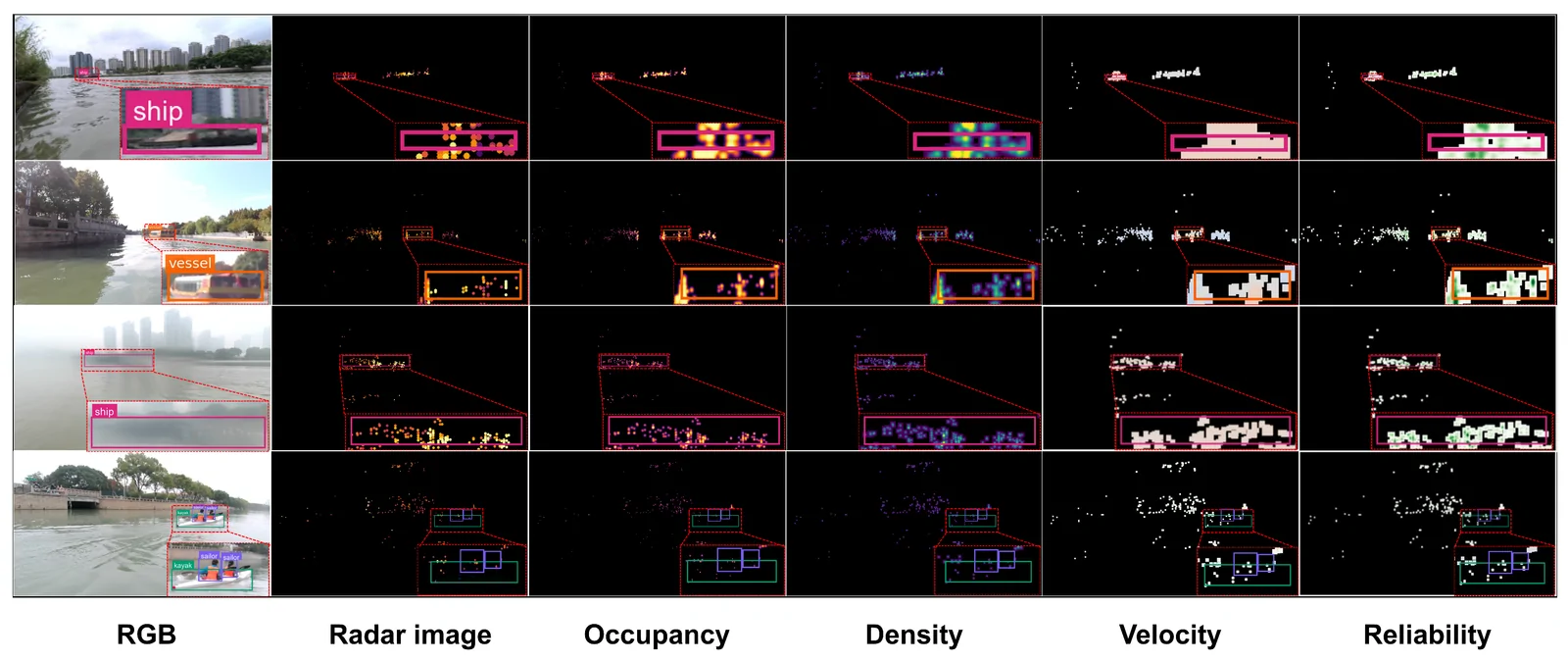
Visualization of the generated probabilistic radar priors. From left to right, the columns show the RGB image, projected radar observations, occupancy prior, density prior, velocity prior, and reliability prior. Bounding-box colors correspond to object categories as follows: red indicates ship, orange indicates vessel, green indicates kayak, and purple indicates sailor.

By decomposing heterogeneous radar measurements into structured spatial, motion, and reliability cues, this stage directly addresses the radar-representation limitation discussed in Section 2.2 and provides a more interpretable basis for subsequent multimodal fusion. As shown in Figure 2, the generated radar priors provide complementary representations of the projected radar observations. The occupancy and density priors describe the spatial existence and local aggregation of radar echoes, respectively. The velocity prior preserves Doppler motion, and the reliability prior reflects observation confidence based on echo intensity, range attenuation, and temporal quality.

### 3.3. Camera-Centric Primary-Auxiliary Feature Extraction

As discussed in Section 2.1 and Section 2.3, visual information remains the primary source of appearance and category-level semantics, although its quality may degrade under challenging water-surface conditions. Radar therefore serves as a complementary source rather than an equally dominant modality. Based on this observation, RPT-Fusion adopts a camera-centric primary-auxiliary architecture that preserves the continuity of the visual pathway while regulating radar contribution through explicit quality control.

For brevity, the camera image $I_{t_{c}}$ is denoted as *I*. The probabilistic radar representation consists of four channels, namely the occupancy prior $M_{\mathrm{occ}}$, density prior $M_{\mathrm{den}}$, velocity prior $M_{\mathrm{vel}}$, and reliability prior $M_{\mathrm{rel}}$. In addition, the scalar temporal quality score $q_{t}$ is spatially broadcast over the input domain Ω to form a constant temporal quality map, i.e., $M_{t}\left(x,y\right)=q_{t}$ for all (x,y)∈Ω. The resulting five-channel radar input is

(9) $$M=\left[M_{\mathrm{occ}},M_{\mathrm{den}},M_{\mathrm{vel}},M_{\mathrm{rel}},M_{t}\right]$$

where the first four channels encode structured radar information and $M_{t}$ introduces frame-level camera–radar temporal consistency into the auxiliary radar branch.

The camera and radar branches subsequently extract paired multi-scale features, denoted by $F_{c}^{s}=\phi_{c}^{s}\left(I\right)$ and $F_{r}^{s}=\phi_{r}^{s}\left(M\right)$, respectively, where s∈{P2,P3,P4,P5}. The two streams are not directly treated as equally important modalities. Instead, the camera features form the primary semantic pathway, whereas radar features are introduced as auxiliary information and are subsequently regulated by the quality-adaptive fusion (QAFusion) mechanism described in Section 3.5. The radar-derived quality terms do not directly estimate visual degradation such as fog, glare, or low illumination. Instead, radar provides complementary geometric and motion cues when visual appearance becomes less discriminative, while the proposed quality mechanism determines whether the radar information itself is sufficiently reliable to contribute to the camera-dominant representation.

### 3.4. Time-Lag-Aware Quality Modeling

As identified in Section 2.4, spatial calibration alone does not guarantee cross-modal consistency in dynamic environments. Even with accurate radar–camera extrinsic calibration, differences between camera and radar acquisition timestamps allow target motion, platform motion, or both to alter the scene state. Consequently, a radar observation that is geometrically valid at the radar acquisition time may become less consistent with the corresponding visual region as the temporal separation increases.

Intuitively, a larger camera–radar sampling interval increases the likelihood that a radar observation projected according to the radar timestamp becomes spatially misaligned with the corresponding object region in the camera frame. Therefore, temporal inconsistency should not be treated merely as a timestamp difference; it directly affects the spatial reliability of the projected radar information.

Rather than treating this temporal difference merely as a synchronization artifact or an additional input value, RPT-Fusion explicitly models it as a reliability variable that affects both radar prior construction and feature-level fusion. Given the camera timestamp $t_{c}$ and radar timestamp $t_{r}$, the temporal offset is $\Delta t=t_{r}-t_{c}$. Its magnitude is clipped as $\bar{\Delta t}=\min(|\Delta t|,\tau_{t})$, where $\tau_{t}$ denotes the maximum temporal offset considered by the model. The corresponding normalized temporal offset is defined as $\delta_{t}=\bar{\Delta t}/\tau_{t}$.

The temporal quality score is modeled using exponential decay, as follows:

(10) $$q_{t}=\exp\left(-\beta_{t}\bar{\Delta t}\right)$$

where $\beta_{t}$ controls the sensitivity of radar reliability to temporal inconsistency. A small temporal offset produces a $q_{t}$ value close to one, whereas increasing temporal separation progressively reduces the reliability assigned to the asynchronous radar observation. In this sense, $q_{t}$ acts as a temporal penalty: radar observations acquired close to the camera timestamp retain stronger reliability, whereas observations with larger temporal offsets are progressively down-weighted because their projected locations are more likely to be inconsistent with the current visual scene.

At the prior level, temporal inconsistency first affects the spatial uncertainty of the projected radar measurements. Let the base anisotropic covariance of the *i*-th radar observation be

$$\Sigma_{i}^{0}=\mathrm{diag}\left({\left(\sigma_{u,i}^{0}\right)}^{2},{\left(\sigma_{v,i}^{0}\right)}^{2}\right)$$

where $\sigma_{u,i}^{0}$ and $\sigma_{v,i}^{0}$ are the range-dependent horizontal and vertical uncertainty scales introduced in Section 3.2. Using the temporal expansion factor $\gamma_{t}=1+\alpha_{t}\delta_{t}$, the covariance is adjusted as

(11) $$\Sigma_{i}^{t}=\gamma_{t}^{2}\Sigma_{i}^{0}={\left(1+\alpha_{t}\delta_{t}\right)}^{2}\Sigma_{i}^{0}$$

where $\alpha_{t}$ controls the rate at which temporal inconsistency enlarges the spatial uncertainty. This formulation increases both horizontal and vertical uncertainty while preserving the anisotropic structure of the radar kernel. Accordingly, a larger temporal offset does not force the model to assume an exact motion-induced displacement; instead, it expands the spatial support of the radar observation to reflect the increased uncertainty of its projected location. Temporal inconsistency simultaneously attenuates radar confidence. Given the intrinsic reliability ${\hat{w}}_{i}^{\mathrm{rel}}$ derived from radar echo strength and sensing distance, the temporally adjusted point reliability is

(12) $$w_{i}^{t}=q_{t}{\hat{w}}_{i}^{\mathrm{rel}}$$

Thus, the measured time lag produces two complementary effects at the prior level: $\delta_{t}$ controls spatial uncertainty expansion through $\Sigma_{i}^{t}$, while $q_{t}$ controls reliability attenuation through $w_{i}^{t}$. The former reflects an increasing uncertainty to which an asynchronous radar response should spatially contribute, whereas the latter reduces how strongly that potentially misaligned response should influence the radar prior.

The final temporally adjusted anisotropic spatial support is expressed as

(13) $$K_{i}^{t}\left(z\right)=\exp\left[-\frac{1}{2}{\left(z-\mu_{i}\right)}^{T}{\left(\Sigma_{i}^{t}\right)}^{-1}\left(z-\mu_{i}\right)\right]$$

where $z={\left[x,y\right]}^{T}$ denotes an image-plane location and $\mu_{i}={\left[u_{i},v_{i}\right]}^{T}$ denotes the projected center of the *i*-th radar observation. A larger camera–radar time lag therefore produces a less spatially concentrated radar response without assuming an exact deterministic displacement. The temporally adjusted reliability prior is then constructed using $w_{i}^{t}$ and $K_{i}^{t}$, namely

(14) $$M_{\mathrm{rel}}\left(z\right)=1-\exp\left(-\sum_{i=1}^{N}w_{i}^{t}K_{i}^{t}\left(z\right)\right)$$

Through the joint action of spatial uncertainty expansion and reliability attenuation, asynchronous radar observations are neither abruptly discarded nor treated as equally reliable compared to synchronized observations. Instead, their spatial responses become less concentrated and their effective contributions are progressively reduced as the temporal offset increases.

At the feature level, the same temporal quality score $q_{t}$ is further transformed into a scale-specific temporal quality term

(15) $$Q_{t}^{s}=\psi_{t}^{s}\left(q_{t}\right),\;s\in \left\{P2,P3,P4,P5\right\}$$

where $\psi_{t}^{s}\left(\cdot \right)$ denotes the temporal projection at feature scale *s*. The resulting $Q_{t}^{s}$ is subsequently combined with the local and global radar quality terms to generate the radar contribution gate described in Section 3.5. Therefore, the proposed time-lag-aware mechanism establishes a dual-level reliability-control process. At the prior level, the measured temporal offset modifies both radar spatial uncertainty and observation reliability through $\Sigma_{i}^{t}$ and $w_{i}^{t}$. At the feature level, $q_{t}$ is propagated through both the temporal quality map $M_{t}$ and the scale-specific quality term $Q_{t}^{s}$. In this way, temporal information continuously regulates radar utilization rather than serving as an isolated auxiliary input.

**Figure 3:**
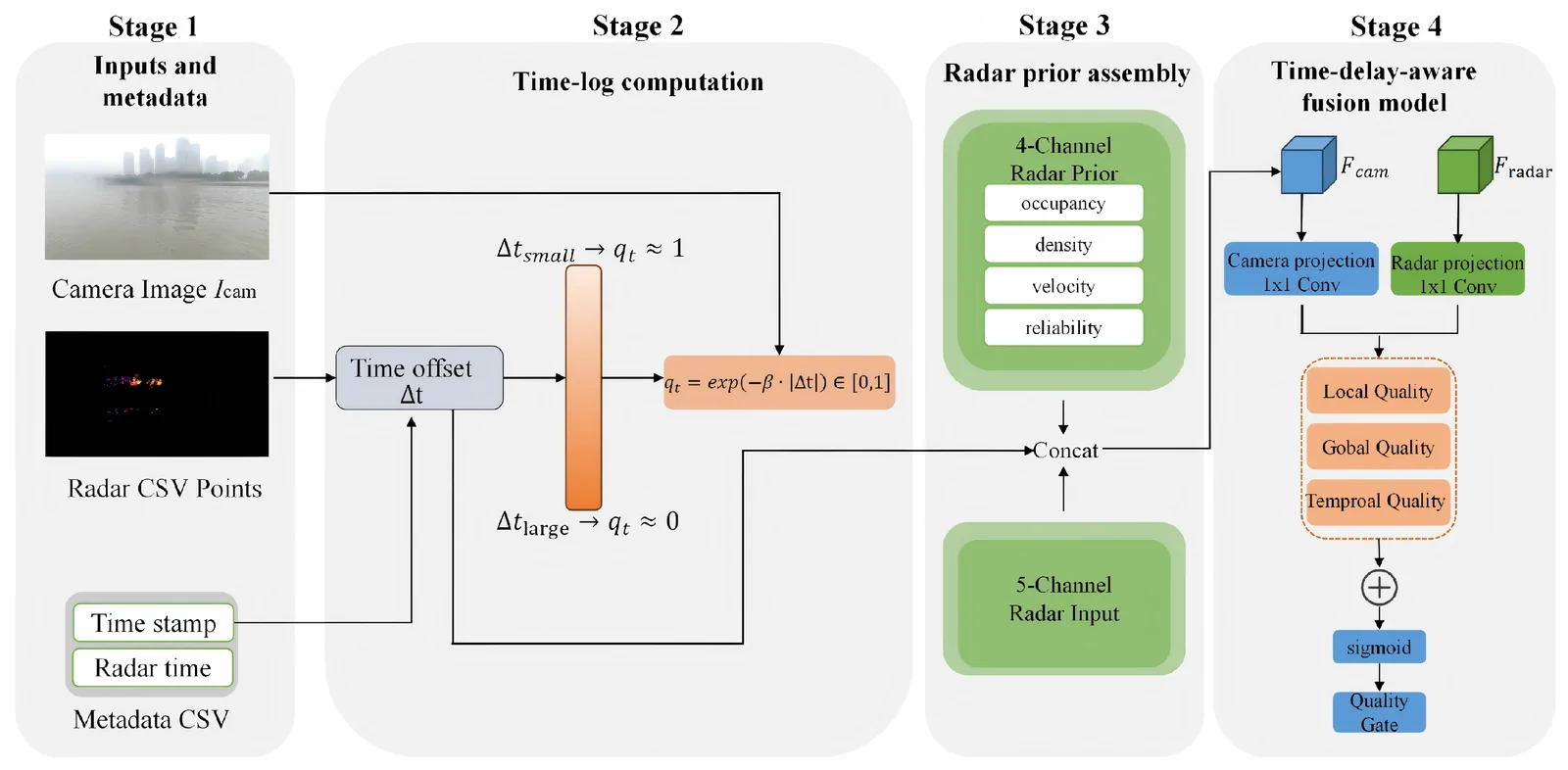
Flowchart of the proposed time-lag-aware quality modeling. The camera and radar timestamps are used to derive temporal uncertainty and reliability information for radar prior construction and feature-level quality-adaptive fusion.

The corresponding time-lag-aware quality modeling process is illustrated in Figure 3.

From an intuitive perspective, this dual-level design converts asynchronous sampling into an explicit reliability-control signal: the larger the camera–radar time gap, the less certain the projected radar location becomes and the less strongly the corresponding radar information is allowed to affect multimodal perception. The proposed mechanism does not explicitly estimate the exact motion-induced displacement of individual radar points. Instead, it models the increased uncertainty associated with asynchronous sensing, thereby avoiding unsupported motion assumptions while preserving a direct connection between measured time lag and radar reliability.

### 3.5. Local–Global–Temporal Quality-Adaptive Fusion

As discussed in Section 2.3, the usefulness of radar information can vary substantially across spatial locations and sensing conditions. Reliable target echoes and strong clutter may coexist within the same radar frame, making uniform or scene-level-only radar weighting insufficient. To explicitly regulate auxiliary radar contribution, RPT-Fusion evaluates radar features from local, global, and temporal perspectives before cross-modal fusion.

For each feature scale *s*, the local and global radar quality terms are estimated as

(16) $$Q_{l}^{s}=\psi_{l}^{s}\left(F_{r}^{s}\right),\;Q_{g}^{s}=\psi_{g}^{s}\left(\mathrm{GAP}(F_{r}^{s})\right)$$

where $Q_{l}^{s}$ captures spatially varying radar quality and $Q_{g}^{s}$ represents the complementary scene-level radar context. The local branch preserves spatial information from $F_{r}^{s}$, allowing different regions to receive different reliability estimates, whereas the global branch summarizes the overall radar feature state through global average pooling. Together with the temporal quality term $Q_{t}^{s}$ defined in Equation (15), the final radar contribution gate is

(17) $$G_{s}=\sigma \left(Q_{l}^{s}+Q_{g}^{s}+Q_{t}^{s}\right)$$

where σ(·) denotes the sigmoid activation and $G_{s}\in \left[0,1\right]$ is the scale-specific radar quality gate. The local, global, and temporal terms play complementary roles in the gating process. The local term $Q_{l}^{s}$ captures spatially heterogeneous radar reliability and determines where radar responses are trustworthy, enabling the model to distinguish useful target-related responses from unreliable local regions. The global term $Q_{g}^{s}$ provides complementary context regarding the overall radar feature state. Importantly, $Q_{g}^{s}$ is not interpreted as a uniform confidence score assigned to all radar observations within a frame. Since reliable target echoes and strong clutter can coexist in complex water-surface scenes, spatial heterogeneity is primarily modeled by $Q_{l}^{s}$, whereas $Q_{g}^{s}$ provides scene-level context without overriding local spatial discrimination.

The temporal term $Q_{t}^{s}$ further introduces cross-sensor temporal consistency into the same contribution-control process. By jointly combining $Q_{l}^{s}$, $Q_{g}^{s}$, and $Q_{t}^{s}$, the gate $G_{s}$ provides scale-specific and spatially adaptive modulation of radar contribution rather than uniformly weighting the entire radar feature map. In this way, local quality determines where radar information is reliable, global quality provides scene-level contextual modulation, and temporal quality accounts for the reliability associated with camera–radar temporal consistency. The gated radar feature is defined as

(18) $${\tilde{F}}_{r}^{s}=G_{s}⊙P_{r}\left(F_{r}^{s}\right)$$

where $P_{r}\left(\cdot \right)$ denotes radar feature projection and ⊙ represents element-wise multiplication. Since the gate is applied only to the radar pathway, $G_{s}$ directly controls the extent to which auxiliary radar information contributes to the subsequent multimodal representation. Radar responses assigned lower-quality values are suppressed, whereas reliable responses retain stronger contributions. The final fused representation is obtained as

(19) $$F_{\mathrm{fuse}}^{s}=\mathrm{Mix}\left(\left[P_{c}\left(F_{c}^{s}\right),{\tilde{F}}_{r}^{s}\right]\right)$$

where $P_{c}\left(\cdot \right)$ denotes camera feature projection and Mix(·) performs cross-modal feature integration.

**Figure 4:**
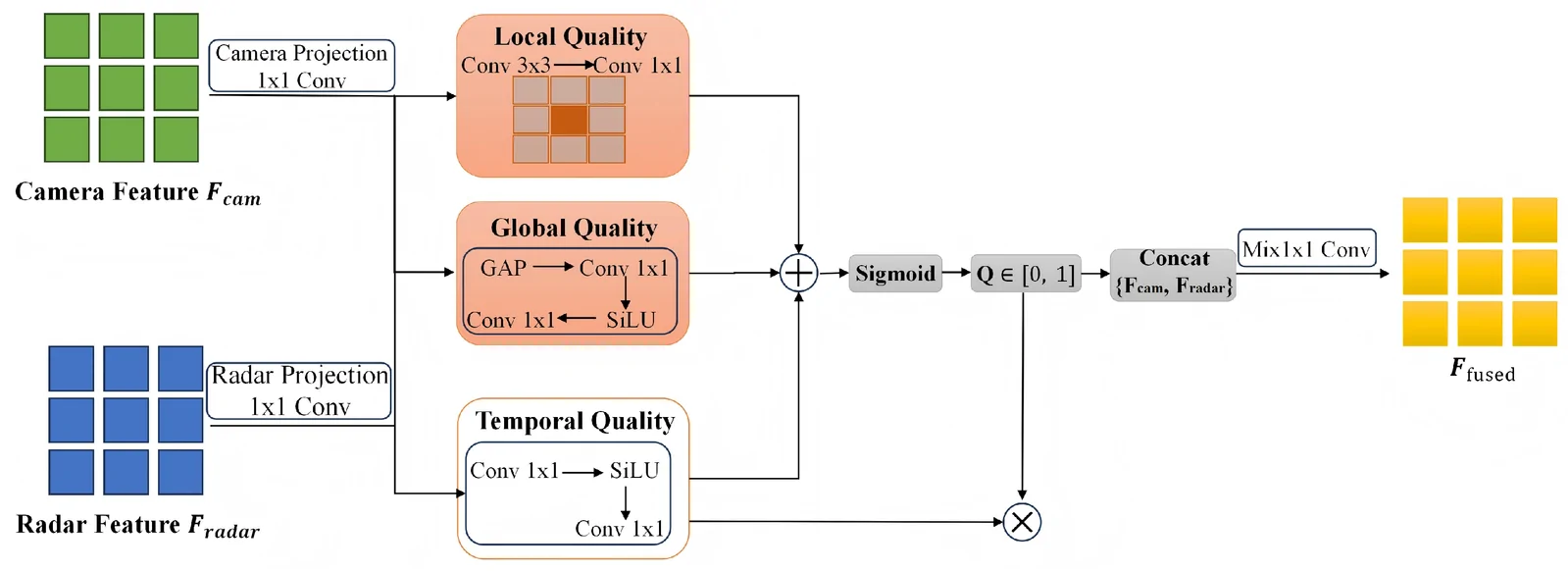
Local–global–temporal quality-adaptive fusion. Local, global, and temporal quality terms jointly generate the radar gate $G_{s}$, which selectively regulates auxiliary radar features before fusion with the primary camera representation.

This asymmetric fusion design directly reflects the camera-centric principle introduced in Section 1. The camera pathway retains direct access to the fused representation, whereas the radar pathway must pass through the learned quality gate before contributing to the detector. Consequently, reliable radar information can complement degraded visual features, while unreliable or temporally inconsistent radar responses are selectively suppressed without interrupting the primary visual pathway. Overall, RPT-Fusion forms a progressive reliability-control chain from radar prior representation and time-lag-aware reliability modeling to feature-level contribution control, enabling complementary radar information to be exploited while preserving robust visual semantics under complex water-surface conditions. The overall local–global–temporal quality-adaptive fusion process is illustrated in Figure 4.

## 4. Experiments

### 4.1. Experimental Setup

Experiments are conducted on the WaterScenes object detection benchmark, which provides RGB images, 4D millimeter-wave radar measurements, timestamps, and object annotations. The seven detection categories are pier, buoy, sailor, ship, boat, vessel, and kayak. The official training, validation, and test split method is adopted. The physical specifications of the radar and camera sensors used in the WaterScenes acquisition platform are summarized in Table 1. Image keyframes are extracted at 2 Hz, while radar measurements are associated with the nearest image timestamp with a maximum tolerated temporal difference of 0.05 s. The raw 4D radar measurements are subsequently converted into the probabilistic radar priors described in Section 3.2 before being fed into the auxiliary radar branch.

RPT-Fusion is implemented based on a YOLO11-style detector. The model employs a custom dual-branch backbone, WaterScenesRadarBackbone, in which the camera and radar inputs are encoded separately and progressively fused through quality-adaptive multi-scale fusion blocks. The detection head follows the YOLO11 P3/P4/P5 detection architecture. The multimodal input comprises eight channels in total. The camera branch receives the three-channel RGB image, whereas the auxiliary radar branch receives a five-channel representation consisting of four probabilistic radar prior channels and one temporal quality channel.

All input images are resized to 640×640, and the model is trained for 300 epochs. The model is trained from scratch without external pretrained weights. The automatic optimizer selection provided by Ultralytics is adopted, which selects MuSGD with an initial learning rate of 0.01 and a momentum of 0.9. The weight decay is set to 0.0005. The nominal batch-size parameter is set to 64, while the actual training batch size is 20. Mixed-precision training is enabled, and 16 dataloader workers are used. During training, geometric data augmentation includes random horizontal flipping with a probability of 0.5, random scaling with factors sampled from [0.5,1.5], translation of up to 10% along each image dimension, and Mosaic augmentation. Mosaic is applied during the first 290 epochs and disabled during the final 10 epochs. During these final 10 epochs, which serve as a fine-tuning stage within the total 300-epoch training schedule, the scaling range is reduced to [0.8,1.2], while horizontal flipping and translation are retained. The same geometric transformations are synchronously applied to the camera images, radar prior maps, and corresponding bounding boxes. No photometric augmentation is applied to the multi-channel fusion inputs.

To alleviate the long-tailed category distribution in WaterScenes, image-level balanced sampling is adopted during training to increase the sampling frequency of tail categories, including buoy, sailor, and kayak. All experiments are conducted on a single NVIDIA RTX 4090 graphics processing unit (GPU) using CUDA 12.1 and PyTorch 2.5.1.

For anisotropic radar prior construction, the horizontal and vertical base scales are set to $\sigma_{u,b}=1.4$ and $\sigma_{v,b}=1.0$, while the corresponding range-dependent coefficients are $\alpha_{u}=2.4$ and $\alpha_{v}=1.6$. Accordingly, $\sigma_{u,i}^{0}=1.4+2.4{\tilde{r}}_{i}$ and $\sigma_{v,i}^{0}=1.0+1.6{\tilde{r}}_{i}$. The parameters are configured such that the mean horizontal–vertical diffusion scale remains equal to the original isotropic formulation $1.2+2.0{\tilde{r}}_{i}$, thereby introducing directional uncertainty without substantially changing the overall diffusion magnitude.

For quantitative evaluation, Precision, Recall, mAP50, and mAP50–95 are used to characterize detection performance from complementary perspectives. Precision measures the proportion of correctly detected objects among all predicted positive detections, while Recall measures the proportion of correctly detected objects among all ground-truth objects. They are defined as

(20) $$\mathrm{Precision}=\frac{TP}{TP+FP}$$

(21) $$\mathrm{Recall}=\frac{TP}{TP+FN}$$

where TP, FP, and FN denote the number of true-positive, false-positive, and false-negative detections, respectively. Precision reflects the correctness of the predicted detections, whereas Recall characterizes the completeness of object retrieval. mAP50 is the mean Average Precision over all object categories when a predicted bounding box is regarded as a correct detection at an Intersection-over-Union (IoU) threshold of 0.50. It is defined as

(22) $$\mathrm{mAP}50=\frac{1}{C}\sum_{c=1}^{C}\int_{0}^{1}P_{c}\left(R\right)\;dR$$

where *C* denotes the number of object categories and $P_{c}\left(R\right)$ denotes the precision–recall curve of category *c* evaluated at an IoU threshold of 0.50. mAP50 therefore measures the overall detection capability under the conventional IoU criterion. mAP50–95 provides a stricter evaluation by averaging the mean Average Precision over ten IoU thresholds from 0.50 to 0.95 with a step size of 0.05, as follows:

(23) $$\mathrm{mAP}50–95=\frac{1}{10C}\sum_{\tau \in \{0.50,0.55,\ldots ,0.95\}}\sum_{c=1}^{C}\int_{0}^{1}P_{c}^{\left(\tau \right)}\left(R\right)\;dR$$

where $P_{c}^{\left(\tau \right)}\left(R\right)$ denotes the Precision–Recall curve of category *c* under IoU threshold τ. Compared with mAP50, mAP50–95 imposes progressively stricter overlap requirements and therefore provides a more comprehensive assessment of both detection and bounding-box localization quality. Using mAP50 and mAP50–95 jointly is particularly relevant to radar–camera fusion because inaccurate or unreliable radar projections may affect not only whether an object is detected but also the spatial accuracy of the predicted bounding box. Precision and Recall are additionally reported in the radar-frame-dropping experiment to evaluate detection correctness and completeness when radar observations are partially or completely unavailable.

### 4.2. Comparison with Existing Methods

RPT-Fusion is compared with representative camera-based detectors and radar–camera fusion methods on WaterScenes. Per-class performance is evaluated using mAP50, while the overall detection performance is assessed using both mAP50 and mAP50–95. All methods in Table 2 are reproduced in our experimental environment and evaluated on the official WaterScenes test set. The same official data split and evaluation protocol are adopted for all compared methods. To reduce the influence of training randomness, each method is independently trained three times, and the overall mAP50 and mAP50–95 are reported as mean ± standard deviation.

At the category level, the per-class mAP50 values of RPT-Fusion are 94.84% for boat, 81.81% for kayak, 92.45% for sailor, 98.27% for vessel, 97.64% for ship, 89.54% for buoy, and 92.88% for pier, as shown in Table 2. RPT-Fusion ranks among the top three methods across all seven categories, indicating consistently competitive detection performance across diverse water-surface object categories in addition to its leading overall performance. RPT-Fusion achieves an average mAP50 of 92.31% and an average mAP50–95 of 68.23% on the official WaterScenes test set, ranking first among all compared methods in both overall metrics. Compared with WS-DETR, which achieves 91.49% mAP50 and 64.54% mAP50–95, RPT-Fusion improves the mean performance by 0.82 and 3.69 percentage points, respectively. Compared with Naive RGB-Radar fusion, which achieves 90.40% mAP50 and 64.71% mAP50–95, the corresponding improvements are 1.91 and 3.52 percentage points.

### 4.3. Ablation Study

Unless otherwise stated, all ablation and robustness experiments in this section are conducted on the official WaterScenes test set using the same 640×640 input resolution, training schedule, preprocessing procedure, and image-level balanced sampling strategy described in Section 4.1. Only the investigated component or evaluation condition is changed within each controlled experiment.

#### 4.3.1. Core Component Ablation Study

A module-level ablation study is conducted to evaluate the contributions of the radar priors, quality-adaptive fusion, and time-lag-aware quality modeling. B0 denotes the RGB-only baseline. B1 introduces the structured radar prior with direct RGB-radar fusion. B2 employs local and global quality estimation while removing temporal quality from the fusion gate. For brevity, this configuration is referred to as QAFusion w/o Temporal Quality throughout the following experiments. B3 introduces temporal quality as an additional radar input without local–global–temporal quality-adaptive fusion. B4 represents the complete RPT-Fusion framework.

As shown in Table 3, the RGB-only baseline achieves 89.29% mAP50. Introducing the structured radar prior through Naive RGB-Radar fusion increases mAP50 to 90.40%, corresponding to an improvement of 1.11 percentage points. Incorporating local and global radar quality estimation further increases mAP50 to 91.96% for QAFusion w/o Temporal Quality. Temporal Input Only achieves 89.87%, whereas the complete RPT-Fusion obtains the highest mAP50 of 92.31%.

Compared with Naive RGB-Radar fusion, QAFusion w/o Temporal Quality improves mAP50 by 1.56 percentage points, indicating the effectiveness of reliability-aware radar contribution control. Further incorporating temporal quality into the local–global quality-adaptive fusion mechanism increases mAP50 from 91.96% to 92.31%, corresponding to an additional gain of 0.35 percentage points. These results indicate that structured radar information and quality-adaptive fusion provide the primary performance gains, while time-lag-aware temporal quality provides a further complementary improvement.

#### 4.3.2. Effect of Anisotropic Radar Prior Modeling

To evaluate the effect of direction-dependent radar uncertainty modeling, the original isotropic Gaussian prior and the proposed range-dependent anisotropic formulation are compared on the official WaterScenes test set under otherwise identical settings.

As shown in Table 4, the isotropic Gaussian formulation achieves 92.03% Precision, 88.07% Recall, 92.01% mAP50, and 68.02% mAP50–95. The anisotropic formulation increases these metrics to 92.21%, 88.25%, 92.31%, and 68.23%, respectively. In particular, mAP50 and mAP50–95 improve by 0.30 and 0.21 percentage points. These results indicate that explicitly modeling direction-dependent radar uncertainty provides a modest but consistent improvement over isotropic radar prior construction.

#### 4.3.3. Time-Lag-Aware Quality Modeling Ablation Study

To evaluate performance under different camera–radar temporal offsets, the official WaterScenes test set is divided according to the absolute camera–radar time difference |Δt| into four intervals: 0–5 ms, 5–10 ms, 10–20 ms, and ≥20 ms. QAFusion w/o Temporal Quality and the complete RPT-Fusion are evaluated under identical settings.

The four time-lag intervals contain 1707, 795, 1568, and 1342 test samples, accounting for 31.54%, 14.69%, 28.97%, and 24.80% of the test set, respectively. The samples are therefore distributed across all four temporal-offset ranges, with approximately one quarter of the test samples exhibiting a camera–radar time difference of at least 20 ms.

As shown in Table 5, QAFusion w/o Temporal Quality achieves mAP50 values of 90.84%, 92.47%, 91.39%, and 92.56% across the four time-lag intervals, whereas RPT-Fusion achieves 91.55%, 92.51%, 91.30%, and 93.71%, respectively. The corresponding changes are +0.71, +0.04, −0.09, and +1.15 percentage points. The largest improvement is observed in the ≥20 ms interval, where temporal-quality modeling improves mAP50 by 1.15 percentage points. In contrast, the intermediate 5–10 ms and 10–20 ms ranges exhibit only marginal changes, indicating that the contribution of temporal-quality modeling is condition-dependent and becomes more evident under relatively large camera–radar temporal offsets.

#### 4.3.4. Robustness to Radar-Frame-Dropping

To evaluate robustness under incomplete radar observations, radar inputs are randomly dropped during evaluation at ratios ranging from 0% to 100%, while the corresponding camera inputs remain unchanged. All settings are evaluated on the official WaterScenes test set using the same trained RPT-Fusion model and evaluation protocol.

As shown in Table 6, the complete-input setting achieves 92.31% mAP50 and 68.23% mAP50–95. As the radar-frame-dropping ratio increases, mAP50 gradually decreases to 91.54% at 100% radar dropping, while mAP50–95 decreases to 67.50%. The corresponding reductions from 0% to 100% radar dropping are 0.77 and 0.73 percentage points, respectively. Precision decreases from 92.11% to 90.96%, whereas Recall changes from 88.25% to 87.27%.

### 4.4. Qualitative Results

**Figure 5:**
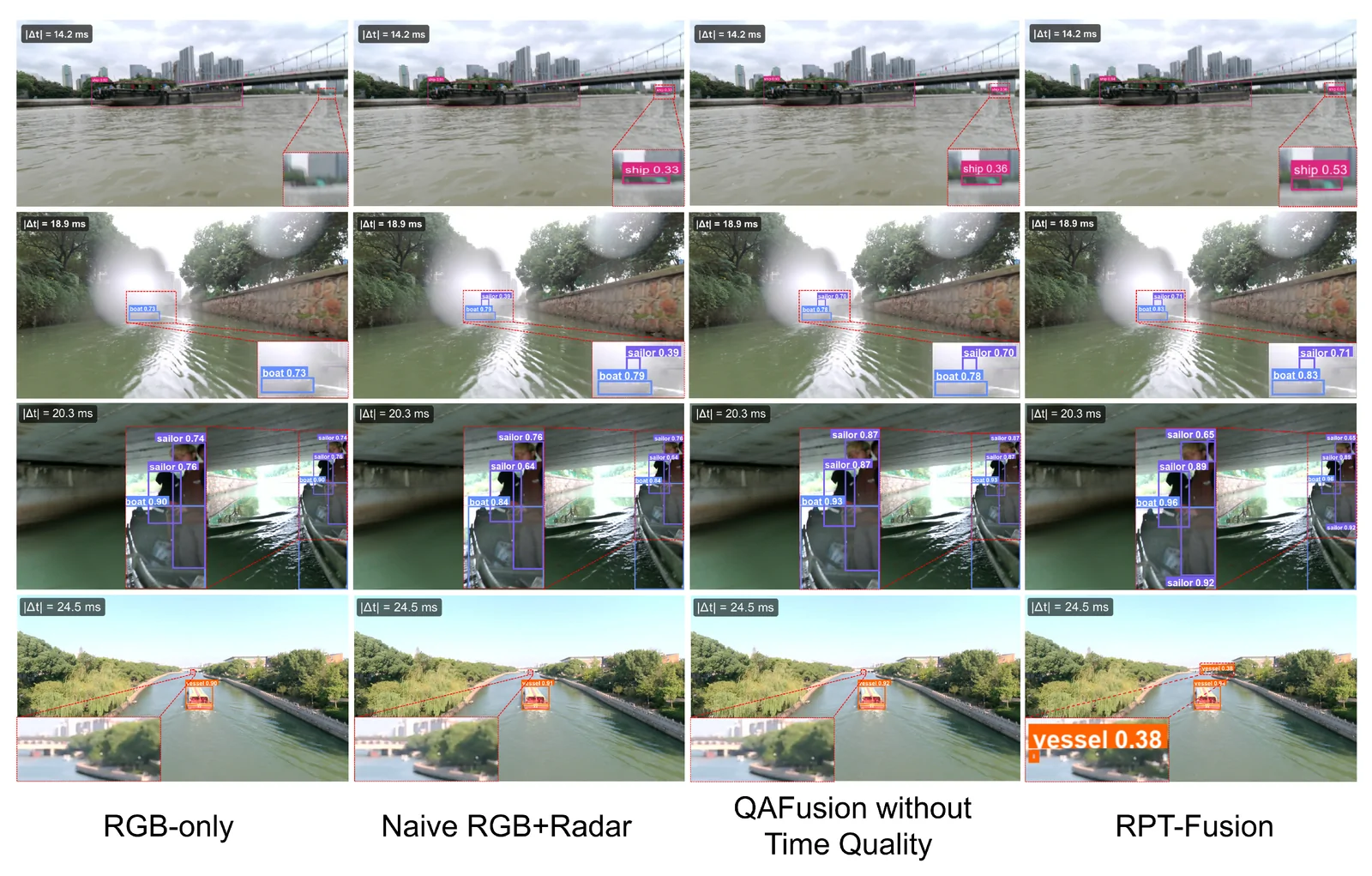
Qualitative detection comparison under challenging water-surface conditions. From left to right, the columns correspond to RGB-only, Naive RGB-Radar, QAFusion w/o Temporal Quality, and RPT-Fusion. Bounding-box colors correspond to object categories as follows: red indicates ship, blue indicates boat, purple indicates sailor, and orange indicates vessel.

Figure 5 compares RGB-only, Naive RGB-Radar, QAFusion w/o Temporal Quality, and the complete RPT-Fusion under representative challenging water-surface conditions. As illustrated in Figure 5, the four configurations produce different detection results across scenes involving distant targets, small targets, reflections, blur, fog, and complex water-surface backgrounds. Compared with RGB-only and Naive RGB-Radar fusion, QAFusion w/o Temporal Quality and RPT-Fusion recover additional targets in several representative examples. The complete RPT-Fusion produces more complete detections in a number of scenes containing weak target appearance and environmental interference.

## 5. Discussion

The experimental results provide further insight into the design choices of RPT-Fusion and their roles in radar–camera object detection in complex water-surface environments. The central principle of the proposed framework is to use the camera as the primary semantic modality while introducing radar as a reliability-controlled auxiliary source. This design differs from direct multimodal fusion, in which radar features are incorporated without explicitly considering their spatial uncertainty or reliability. As shown in Table 2, RPT-Fusion achieves 92.31% mAP50 and 68.23% mAP50–95 on the official WaterScenes test set, outperforming both Naive RGB-Radar fusion and the representative radar–camera detector WS-DETR in overall performance. This improvement is more pronounced under mAP50–95, indicating that the proposed reliability-controlled fusion strategy is particularly beneficial when stricter localization criteria are considered.

The component ablation results in Table 3 further clarify where this improvement originates. Introducing the radar prior increases mAP50 from 89.29% for the RGB-only baseline to 90.40%, showing that radar measurements provide complementary information beyond visual appearance alone. However, directly introducing radar information does not fully exploit this complementarity. When local and global quality estimation is incorporated, mAP50 further increases to 91.96%, while the complete RPT-Fusion reaches 92.31%. These results support the use of an asymmetric primary-auxiliary architecture in which radar contribution is explicitly regulated rather than unconditionally injected into the visual representation. In particular, QAFusion w/o Temporal Quality improves mAP50 by 1.56 percentage points over Naive RGB-Radar fusion, while introducing temporal quality into the complete RPT-Fusion further provides a 0.35 percentage-point improvement. This indicates that quality-adaptive radar contribution control provides the major performance gain, while temporal reliability modeling offers an additional complementary benefit.

The probabilistic radar prior also plays an important role in converting sparse radar observations into an image-aligned representation suitable for feature fusion. In particular, the anisotropic Gaussian experiment in Table 4 shows that replacing isotropic diffusion with direction-dependent uncertainty modeling improves mAP50 from 92.01% to 92.31% and mAP50–95 from 68.02% to 68.23%. Because the average spatial scale is kept comparable between the two formulations, this improvement is associated with the directional structure of the radar prior rather than simply using a wider spatial kernel. This result supports the use of anisotropic image-plane uncertainty when projecting sparse radar observations into the camera coordinate domain. Nevertheless, the current formulation remains a parameterized approximation of projection uncertainty rather than a complete covariance model derived from radar sensing geometry, calibration uncertainty, and target motion.

Temporal quality provides a second level of reliability control by accounting for asynchronous camera–radar acquisition. The core ablation shows that temporal quality used only as an additional radar input achieves substantially lower performance than the complete local–global–temporal quality formulation. This suggests that the temporal offset is more useful as a reliability-control variable than as an ordinary input feature. The time-lag bucket analysis in Table 5 further reveals that this effect is not uniform across temporal-offset intervals. RPT-Fusion differs only marginally from QAFusion without temporal quality in the 5–10 ms interval and shows a slight decrease in the 10–20 ms interval, whereas the largest gain of 1.15 percentage points is observed for samples with |Δt|≥20 ms. Therefore, the temporal mechanism should not be interpreted as producing a monotonic performance increase with time lag. Instead, its role is condition-dependent: it provides an additional means of regulating radar contribution when temporal inconsistency becomes relevant, with the strongest observed improvement occurring in the largest time-lag subset.

The radar-frame-dropping experiment provides a complementary view of the proposed primary-auxiliary design. As the radar-frame-dropping ratio increases from 0% to 100%, mAP50 decreases from 92.31% to 91.54%, while mAP50–95 decreases from 68.23% to 67.50%. The corresponding reductions are by 0.77 and 0.73 percentage points, respectively. Although performance decreases as radar availability is reduced, the degradation remains limited even when all radar frames are removed at inference time. This behavior is consistent with the camera-centric design of RPT-Fusion, in which the visual pathway remains directly available while radar information serves as a quality-controlled auxiliary source. The radar-frame-dropping experiment therefore characterizes inference-time robustness to incomplete radar observations, while the component ablation results separately demonstrate the performance benefit of reliable radar information when it is available. It should be emphasized that the 100% radar-dropping condition is not equivalent to the independently trained RGB-only baseline in Table 3: the former evaluates an already-trained multimodal RPT-Fusion model with radar removed only during inference, whereas the latter is trained without radar information from the beginning. The two experiments therefore evaluate different aspects of the framework, namely the contribution of multimodal learning and the robustness of the trained model to missing auxiliary observations.

Taken together, the experimental results support a unified reliability-control interpretation of RPT-Fusion. The probabilistic radar prior determines how sparse radar measurements are represented in the image domain; local and global quality estimation determines how strongly radar features contribute at different spatial and scene contexts; and temporal quality further adjusts this contribution according to camera–radar synchronization. These components collectively allow radar to complement visual perception without replacing the camera as the primary semantic source. This property is particularly relevant to water-surface environments, where visual observations may be degraded by reflections, waves, low illumination, rain or fog, complex backgrounds, and weak target appearance, while radar observations may simultaneously be affected by sparsity, clutter, multipath propagation, attenuation, or temporal inconsistency.

Several limitations remain. First, the proposed framework operates on radar observations after point-cloud generation and therefore treats multipath propagation, attenuation, interference, and water-surface clutter mainly through downstream reliability modeling rather than explicitly compensating for these effects at the radar signal-processing level. Second, the anisotropic Gaussian prior is defined in the image plane using parameterized horizontal and vertical uncertainty scales. Although the ablation results support this formulation, the Gaussian prior does not explicitly propagate the complete radar measurement covariance, calibration uncertainty, or perspective-projection uncertainty into the camera image. Third, the temporal quality score is estimated at the frame level from the camera–radar timestamp difference. It therefore models temporal inconsistency globally and does not explicitly estimate object-specific motion or the spatial displacement induced by target motion and ego-motion. Fourth, although the radar-frame-dropping experiment indicates that the camera-centric architecture remains functional under missing radar observations, the contribution of radar still depends on the density and quality of the available returns. Extremely sparse or unreliable radar measurements may therefore limit the benefit of multimodal fusion. Future work will investigate physically grounded radar uncertainty propagation, explicit modeling of radar propagation artifacts, object-level spatiotemporal alignment, and more adaptive reliability estimation under sparse or degraded radar observations.

## 6. Conclusions

This paper presents RPT-Fusion, a radar–camera fusion framework for robust water-surface object detection, in which the camera serves as the primary semantic modality and radar is introduced as a reliability-controlled auxiliary source. Experiments on the WaterScenes dataset show that RPT-Fusion achieves 92.31% mAP50 and 68.23% mAP50–95 on the official test set, outperforming naive RGB-radar fusion and representative existing methods. Ablation experiments further verify the contributions of structured radar prior representation, quality-adaptive fusion, and temporal-quality modeling. The anisotropic radar prior formulation improves mAP50 from 92.01% to 92.31% and mAP50–95 from 68.02% to 68.23% over the isotropic baseline, while the time-lag analysis shows that temporal quality provides condition-dependent benefits, with the largest observed gain of 1.15 percentage points occurring when the camera–radar offset reaches or exceeds 20 ms. Radar-frame-dropping experiments further show that as radar observations are progressively removed, mAP50 decreases from 92.31% to 91.54% and mAP50–95 decreases from 68.23% to 67.50%, indicating that the camera-centric architecture retains a substantial detection capability under incomplete radar observations while still benefiting from reliable radar information when available. Overall, the results support the proposed reliability-control strategy for integrating sparse, uncertain, and temporally inconsistent radar information into a camera-dominant detection framework. Future work will focus on more physically grounded radar uncertainty modeling, object-level spatiotemporal alignment, and improved robustness under sparse or degraded radar observations.

## Figures and Tables

**Table 1:** Specifications of the radar and camera sensors used in WaterScenes [1].

Sensor	Specification	Value
Radar	Model	Oculii EAGLE
	Type	77 GHz 4D point-cloud radar
	Operating mode	Medium-range mode
	Maximum detection range	200 m
	Range resolution	0.43 m
	Velocity resolution	0.27 m/s
	Angular resolution	<1° in azimuth/elevation
	Field of view/capture frequency	$110^{{}^{\circ}}$ HFOV, $45^{{}^{\circ}}$ VFOV/15 Hz
Camera	Sensor model	SONY IMX317 CMOS
	Image resolution	1920×1080
	Field of view	$100^{{}^{\circ}}$ HFOV, $60^{{}^{\circ}}$ VFOV
	Capture frequency	30 Hz

**Table 2:** Performance comparison with existing object detection models on the official WaterScenes test set. In the modalities column, C denotes camera input and R denotes 4D-mmWave radar input. The values reported for boat, kayak, sailor, vessel, ship, buoy, and pier correspond to per-class mAP50. The overall mAP50 and mAP50–95 are reported as mean ± standard deviation over three independent runs. All values are reported in percentages (%). The top three results are highlighted in red, blue, and green, respectively.

Model	Modalities	Boat	Kayak	Sailor	Vessel	Ship	Buoy	Pier	mAP50–95	mAP50
Faster R-CNN [37]	C	88.96	58.27	75.69	92.29	93.03	78.48	81.37	47.80 ± 0.15	81.14 ± 0.01
CenterNet [38]	C	89.58	62.92	79.31	93.15	92.72	80.10	83.04	54.79 ± 0.14	82.92 ± 0.05
DAB-DETR [39]	C	84.55	67.55	53.76	90.71	89.74	55.02	68.74	38.88 ± 0.28	72.88 ± 0.13
DAB-Deformable-DETR [39,40]	C	74.01	70.85	50.97	81.18	70.60	56.04	60.62	47.33 ± 0.20	66.25 ± 0.20
Deformable DETR [40]	C	89.46	66.87	80.26	92.78	92.95	82.21	83.98	56.59 ± 0.17	84.04 ± 0.16
RT-DETR [41]	C	92.38	66.42	87.38	97.51	97.39	88.24	92.90	59.87 ± 0.11	88.92 ± 0.09
DINO [42]	C	91.35	83.81	80.99	95.85	95.17	84.01	89.95	57.87 ± 0.13	88.70 ± 0.03
YOLOX-M [43]	C	89.51	76.15	80.56	92.13	91.47	81.14	85.14	57.88 ± 0.02	85.17 ± 0.01
YOLOv8-M [44]	C	90.87	62.51	82.19	95.88	93.70	84.31	80.69	59.27 ± 0.27	84.42 ± 0.06
YOLOv9-M [45]	C	93.59	66.23	84.61	96.19	94.76	86.35	88.02	61.66 ± 0.21	87.12 ± 0.13
YOLOv10-M [46]	C	93.74	67.44	87.40	97.02	95.68	87.73	88.90	62.91 ± 0.29	88.29 ± 0.29
YOLOv11-M [47]	C	94.36	68.18	87.21	97.17	96.23	88.48	90.08	63.94 ± 0.09	88.90 ± 0.17
YOLOX-M [43]	C + R	91.34	77.12	81.38	92.50	92.94	82.23	85.58	59.50 ± 0.21	86.15 ± 0.25
YOLOv8-M [44]	C + R	91.27	77.97	85.10	95.03	94.69	85.96	86.28	61.27 ± 0.27	88.04 ± 0.25
Naive RGB-Radar	C + R	94.28	77.42	88.07	97.32	96.93	88.99	90.50	64.71 ± 0.08	90.40 ± 0.15
WS-DETR [36]	C + R	92.37	74.72	92.99	98.39	97.83	89.83	94.16	64.54 ± 0.05	91.49 ± 0.02
Ours	C + R	94.84	81.81	92.45	98.27	97.64	89.54	92.88	68.23 ± 0.14	92.31 ± 0.19

**Table 3:** Core component ablation study on the official WaterScenes test set.

Setting	Radar	Time	Quality	mAP50 (%)
B0: RGB-only	×	×	–	89.29
B1: Naive RGB-Radar	✓	×	–	90.40
B2: QAFusion w/o Temporal	✓	×	L + G	91.96
B3: Temporal Input Only	✓	✓	–	89.87
B4: RPT-Fusion	✓	✓	L + G + T	92.31

L, G, and T denote the local, global, and temporal quality terms $Q_{l}^{s}$, $Q_{g}^{s}$, and $Q_{t}^{s}$, respectively.

**Table 4:** Comparison between isotropic and anisotropic radar prior modeling on the official WaterScenes test set. All values are reported in percentages (%).

Radar Prior	Precision	Recall	mAP50	mAP50–95
Isotropic Gaussian	92.03	88.07	92.01	68.02
Anisotropic Gaussian	92.21	88.25	92.31	68.23

**Table 5:** Time-lag bucket analysis on the official WaterScenes test set. All mAP50 values are reported in percentages (%), while the performance differences are reported in percentage points (pp).

|Δt|	Samples	QAFusion w/o Temporal	RPT-Fusion	Gain (pp)
0–5 ms	1707	90.84	91.55	+0.71
5–10 ms	795	92.47	92.51	+0.04
10–20 ms	1568	91.39	91.30	-0.09
≥20 ms	1342	92.56	93.71	+1.15

**Table 6:** Robustness analysis under different radar-frame-dropping ratios using the official WaterScenes test set. The 0% setting corresponds to complete radar availability. All values are reported in percentages (%).

Radar Drop	Precision	Recall	mAP50	mAP50–95
0%	92.11	88.25	92.31	68.23
10%	91.93	87.30	92.19	67.95
25%	91.81	88.05	91.92	67.94
50%	91.60	87.95	91.90	67.87
75%	91.06	87.75	91.78	67.78
100%	90.96	87.27	91.54	67.50

## Data Availability

The data presented in this study are available on request from the corresponding author. The implementation details and experimental settings are described in this manuscript.
